# Carrot Intake and Risk of Colorectal Cancer: A Prospective Cohort Study of 57,053 Danes

**DOI:** 10.3390/nu12020332

**Published:** 2020-01-27

**Authors:** Ulrik Deding, Gunnar Baatrup, Lars Porskjær Christensen, Morten Kobaek-Larsen

**Affiliations:** 1 Department of Clinical Research, University of Southern Denmark, 5000 Odense, Denmark; Ulrik.Deding@rsyd.dk (U.D.); Gunnar.Baatrup@rsyd.dk (G.B.); 2 Department of Surgery, Odense University Hospital, 5000 Odense, Denmark; 3 Department of Chemistry and Bioscience, Faculty of Engineering and Science, Aalborg University, 6700 Esbjerg, Denmark; lpch@bio.aau.dk

**Keywords:** carrots, apiaceous vegetables, colorectal cancer, risk, cohort study

## Abstract

Carrots are consumed worldwide. Several meta-analysis studies on carrot consumption have indicated that carrots play a central role as a protecting vegetable against development of different types of cancers. A cancer-preventive role of carrots is plausible because they are the main dietary source of the bioactive polyacetylenic oxylipins falcarinol (FaOH) and falcarindiol (FaDOH), which have shown anti-proliferative and anti-inflammatory activity in numerous in vitro studies. In addition, purified FaOH and FaDOH have, in recent studies in colorectal cancer (CRC)-primed rats, demonstrated an anti-neoplastic effect in a dose-dependent manner. The mechanisms of action for this effect appears to be due to inhibition of pro-inflammatory and transcription factor biomarkers for inflammation and cancer. However, studies of the CRC-preventive effect of carrots in a large cohort are still missing. We therefore examined the risk of being diagnosed with CRC as predicted by intake of carrots in a Danish population of 57,053 individuals with a long follow-up. Self-reported intake of raw carrots at a baseline of 2–4 carrots or more each week (>32 g/day) was associated with a 17% decrease in risk of CRC with a mean follow-up of >18 years, compared to individuals with no intake of raw carrots even after extensive model adjustments (HR 0.83 CI 95% 0.71; 0.98). An intake below 2–4 carrots each week (<32 g/day) was not significantly associated with reduced risk of CRC (HR 0.93 CI 95% 0.82; 1.06). The results of this prospective cohort study clearly support the results from studies in cancer-primed rats for CRC and hence a CRC-preventive effect of carrots.

## 1. Introduction

The cancer-preventive effects of fruit and vegetables have been intensively studied for over 30 years. According to early epidemiological research, there is an inverse association between the intake of fruit and vegetables and the risk of developing cancers [1,2,3]; however, recent epidemiological studies indicate that for common cancers such as breast, colorectal, lung and prostate cancer, small or no association between total fruit and vegetable consumption and cancer risk exists [4,5]. In early studies, the cancer-preventive effects of fruit and vegetables were mainly ascribed to their content of minerals, fibers and antioxidants. However, fruit and vegetables have very varied composition of nutrients and other constituents, and therefore it is still possible that there are cancer-preventive effects to be identified for the intake of individual fruit and vegetables. In particular, vegetables contain a wide variety of compounds with many interesting bioactivities that are unrelated to antioxidant effects. Some of these bioactive constituents may contribute to the potential cancer-preventive effects of vegetables and thus may help to obtain a deeper understanding of their health promoting effects in general [6,7,8]. This is, for example, the case if we look at apiaceous vegetables such as carrots that are consumed worldwide and in particular in North America and in European countries.

Carrots are rich in carotenoid antioxidants such as β-and α-carotene and epidemiological studies have shown that a high content of β-carotene in the blood is correlated with a low incidence of cancers and other diseases [9,10,11]. In most European countries and North America, more than 50% of the β-carotene intake is provided by carrots, although in these regions of the world, carrot consumption is better correlated with the intake of α-carotene [12]. Several studies have also found stronger negative correlations of developing cancer and in particular lung cancer with intake of α-carotene rather than β-carotene [13,14]. Thus, it is widely accepted that carrots play a central role as a protecting vegetable against development of cancer, which is supported by recent meta-analysis studies on carrot consumption in relation to the development of breast, gastric, lung and prostate cancer [15,16,17,18]. The cancer-preventive effect of this vegetable has mainly been explained by its high contents of carotenoids; however, intervention studies have shown that supplementation with carotenoids does not protect against development of this disease [10,11,19]. Hence, α-carotene and β-carotene may be biomarkers for the intake of other bioactive constituents in carrots with cancer-preventive effects. Such potential anticancer compounds are indeed present in carrots and includes mainly phenylpropanoids [20] and polyacetylenic oxylipins [21,22,23,24,25,26,27,28] of which the latter type of bioactive constituents are the most studied both in vitro and in vivo.

The major polyacetylenic oxylipins in carrots are falcarinol (FaOH) and falcarindiol (FaDOH) and carrots are the major dietary source of this type of bioactive compounds, although they are also present in other apiaceous vegetables such as celery, celeriac, fennel, and parsley [28,29,30]. FaOH and FaDOH have received considerable attention in recent years due to their cytotoxic and anti-inflammatory activities in vitro [21,24,25,26,29,30,31,32,33,34] and, recently, the anti-neoplastic effects of these polyacetylenic oxylipins have been demonstrated in cancer-primed rat models for colorectal cancer (CRC) [21,23,27].

CRC is the third cause of cancer-related death in developed countries and is probably associated with a modern lifestyle typified by limited physical activity, alcohol consumption and dietary changes [35,36]. CRC is a metastatic type of cancer that is developed in a multistep process, from normal epithelial cells via inflammation to aberrant crypt foci and progressive adenoma stages, to carcinomas [37,38]. To reduce the incidence and consequences of CRC, effective prevention and treatment strategies need to be identified. Due to the long precancerous stage of this disease, dietary intervention may exert favorable effects on polyp formation and/or inhibition of adenomas transformation to CRC. Recent findings indicate that long-term consumption of a diet rich in vegetables may prevent the development of CRC [39,40]. Chronic inflammation appears to play key role in the development of CRC. Cyclooxygenase 2 (COX-2) levels are low in normal tissue but are rapidly induced as an early response to growth factors, cytokines and tumor promoters associated with inflammation, abnormal proliferation, angiogenesis, invasion, and metastasis, and the existence of an association between CRC and COX-2 overexpression appears to be established [41,42,43]. Studies on the anti-neoplastic effects of FaOH and FaDOH in tumors of cancer-primed rats for CRC have demonstrated that these polyacetylenic oxylipins inhibit pro-inflammatory and transcription factor biomarkers for inflammation and cancer such as COX-2, interleukin 6 (IL-6), tumor necrosis factor alfa (TNF-α) and nuclear factor kappa-light-chain-enhancer of activated B cells (NF-κB) [21]. Hence, carrots contain bioactive compounds that target CRC development and thus carrot intake could be one of the most important dietary measures for the prevention of CRC. Polyacetylenic oxylipins are, however, sensitive to heat, light and oxidation, and previous investigations of blanched or cooked carrots have shown that the content of these bioactive compounds may be reduced up to 70% by thermal processing [44,45]. Furthermore, carrot juice represents another source of polyacetylenic oxylipins, but the content of these bioactive compounds is also reduced in carrot juice compared to raw carrots due to their low water solubility and possible thermal treatment as well as pH of the carrot juice [46]. On the other hand, the availability of these compounds may be increased in processed vegetables due to alteration of the structure and digestibility of the food thus increasing their bioavailability, which to some extent can offset the cancer-preventive effect of raw versus processed carrots. This is consistent with the fact that no major differences in the cancer-preventive effects of raw versus cooked vegetables, including carrots, have been observed [47].

However, more specific studies of the effect in a human population are still missing in order to conclude whether carrot intake reduces risk of CRC and what quantity of carrot intake is sufficient. The aim of this study was to investigate the risk of being diagnosed with CRC as predicted by intake of carrots in a large Danish study population with a long follow-up.

## 2. Materials and Methods

### 2.1. Study Population

This study was conducted as a prospective cohort study investigating risk of CRC in individuals originally included in the Diet, Cancer and Health study [48]. Follow-up was conducted using Danish National Registers (described in detail below).

In total, 160,725 individuals aged from 50 to 64, who were born in Denmark, with residence in the area of Aarhus or Copenhagen and had no previous cancer diagnose registered were invited for participation. Inclusion for the Diet, Cancer and Health cohort was initiated in 1993 and ended by 1997. Individuals received written information and invitation. In case of non-responders, reminders were distributed three weeks past initial invitation. After an additional three weeks with no response, a new invitation letter was sent. A validated 192 item food frequency questionnaire was filled out by the participants on their consumption of categorized food and beverages during the last 12 months [48,49,50]. Further, the individuals filled in a lifestyle questionnaire collecting data on known cancer risk factors including history of smoking, alcohol, physical activity, previous illness, education and occupation. A lab technician conducted measurements of height and weight on each participating individual [48]. Individuals were followed until 31st of December 2016 or until they were diagnosed with CRC, died or emigrated (and thereby lost for follow-up).

### 2.2. Data from Registries

The Danish Civil Registration System held information on the personal identification number of all individuals with permanent residence in Denmark, as well as dates of migration and dates of death [51]. This made it possible to follow each individual in the National registers in this study. The Danish Cancer Registry records all incidences of malignant neoplasms and some precancerous and benign lesions in the Danish population [52] and was used to identify CRC diagnoses.

### 2.3. Exposure

As carrots are the major dietary source of the bioactive compounds of interest, they were the main exposure in this study. Embedded in the food frequency questionnaire were items regarding raw carrot intake, carrot juice intake and prepared/cooked carrots. As the levels of FaOH and FaDOH decreases significantly when carrots are blanched or cooked, thermal processed carrots are of lesser interest than raw in relation to cancer-preventive measures. The same is the case for carrot juice as described in the introduction. Therefore, raw carrot was chosen as the main exposure, but carrot juice and thermal processed carrots were included in sensitivity analyses. Participants reported frequency of intake ranging from never to 8 or more times per day. Using standard portion sizes, the daily intake in grams was then calculated using FoodCalc [53]. This resulted in 11 different possible values of intake in grams per day (g/day) per individual ranging from 0 to 487.5 g per day. The dose of FaOH and FaDOH needed to identify potential risk reductions in CRC development is unknown. However, recent studies on the effect of FaOH and FaDOH on precursor lesions of CRC in azoymethane-induced rats indicate that a cancer-preventive effect in humans may be obtained with an intake of more than 2.5 mg of each polyacetylene per day [21,23]. The content of FaOH and FaDOH varies considerably between carrot genotypes [45,54,55] but based on the results from the CRC-primed rat studies, it is estimated that the intake of raw carrots should be at least between 30 and 100 g per day to obtain a cancer-preventive dose of polyacetylenes. Although, the cancer preventing effect of FaOH and FaDOH previously seen in rats was a logarithmic dose-response effect [21], it is assumed that the intake of carrots will result in a non-linear effect due to variability in the concentration of these polyacetylenes in different commercially available carrot genotypes. Thus, the exposure variable should be treated categorically. Based on this information and after investigating the distribution of raw carrot intake in the study population and the incidence of CRC in each of the 11 possible g/day groups, a further categorization of no raw carrot intake, less than 32 g/day and more than 32 g/day was performed.

### 2.4. Outcome

Outcome was defined as any CRC diagnosis. CRC diagnoses were identified by ICD-10 codes in the registries. ICD-10 codes identified as CRC were C180–189, C199 and C209.

### 2.5. Covariates

Gender and age were included in model II. Age was divided into three groups of 50–54, 55–59 and 60–65 years of age. Previous cerebral or coronary artery thrombosis and intake of nonsteroidal anti-inflammatory drugs (NSAIDs) were included in model III as dichotomous variables created from self-reported answers to the lifestyle questionnaire. Smoking, alcohol intake, body mass index (BMI) and metabolic equivalents (MET) score were included in model IV and were based on self-reported answers from the lifestyle questionnaire except for BMI, which was calculated based on height and weight, which was measured by a lab technician at baseline and categorized as low (<18.5), normal (18.5–25) and high (>25). Smoking was categorized as non-smoker, former smoker and current smoker. Alcohol intake was categorized as no alcohol intake, intake within current recommendations and intake over the current recommendations. Danish low risk recommendations were at the time of collecting the data from the study population no more than 14 units per week for women and no more than 21 units per week for men but today the recommendations have been changed to <7 units for women and <14 units for men [56]. MET score was calculated from self-reported activity levels in lifestyle questionnaire and categorized as quartiles in the study population. Intake of other root vegetables (celery, ginger root and part frozen carrot from vegetable mix) and intake of all other vegetables (not including root vegetables or carrots) were included in model V and was derived from food frequency questionnaire. Other root vegetables and all other vegetables were categorized as quartiles of intake in the study population.

### 2.6. Ethics

The Diet, Cancer and Health study was originally approved by the relevant scientific committees and the Danish data protection agency. Individual informed consent was obtained from each participant in order to link cohort data with information from national registers [48]. Only aggregated anonymized analyses and results are published in the current study.

### 2.7. Statistical Analysis

*X*^2^-tests were used to compare baseline characteristics. Cumulative incidence proportion curves of CRC diagnoses were created, in which individuals who died or emigrated were censored. As inclusion for the study was done in a period longer than four years, the possible follow-up time varied between individuals. Cumulative incidence proportion curves were therefor limited to 7157 days, which was the shortest possible observation time for individuals alive in Denmark with no CRC diagnosis by 31st December 2016 (from last inclusion day until 31st of December 2016). Cox proportional hazard regression models were conducted testing the correlations between raw carrot intake and incidence of CRC. Model I was a univariate regression model and model II–V were multivariate models, increasingly adjusting for other factors. Other root vegetables and vegetables in general were included in the regression models as covariates to isolate the effect of carrots. Demographic characteristics, lifestyle habits and intake of medication influencing the COX-2 activity were included as covariates in the models as well. Tests for interactions between main exposure variable and each of the included covariates in the full model were conducted. Sensitivity analyses investigating carrot juice, processed carrots and total carrot intake (raw, processed and juice combined) as exposures were also conducted. Sensitivity analysis was conducted using age as time, comparing individuals of the same age instead of adjusting for age group at entry as a covariate in the regression models. Schoenfeld residuals were examined to verify the proportional hazards assumption. Data management was performed using SAS software version 9.4 (SAS Institute Inc. SAS 9.4. Cary, North Carolina, USA) and statistical analyses were conducted using R statistical software package version 3.6.1 (R Core Team, Vienna, Austria) (packages: Publish and Survival) [57,58,59].

## 3. Results

**Figure 1:**
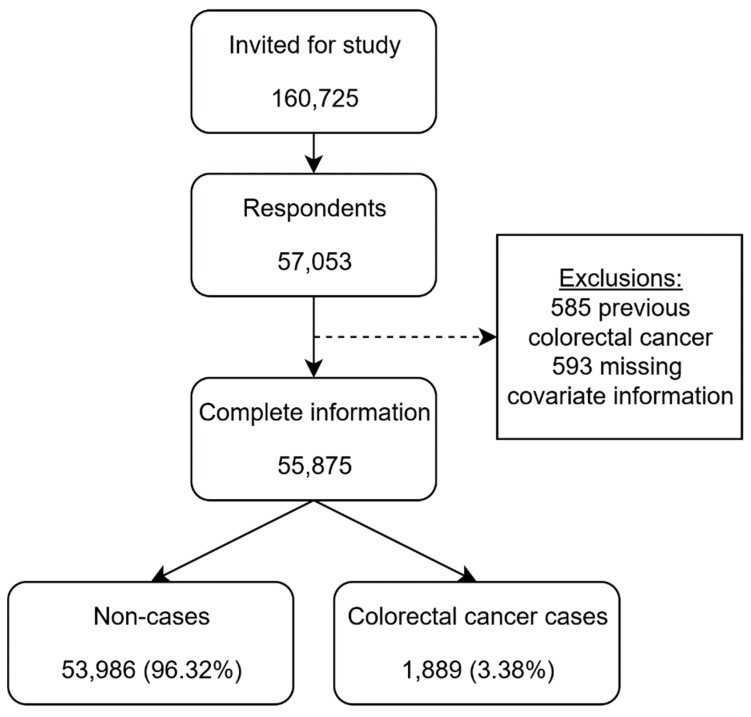
Flow chart of 160,725 individuals invited for participation in “Diet, Cancer and Health”.

Out of 160,725 invited individuals, 57,053 participated in the Diet, Cancer and Health study. In total, 585 individuals were excluded due to previous CRC diagnosis and 593 individuals were excluded due to missing information for covariates. Complete information was obtained for 55,875 individuals who were eligible for analysis. In total, 1889 (3.38%) were diagnosed with CRC during follow-up (Figure 1). Follow-up time varied from 3 to 8438 days, with a mean of 6845 days.

Investigation of the 11 groups of self-reported daily intake of raw carrot and register-based incidence of CRC showed a pattern (adjusted for age and gender), in which groups reporting an intake less than 32 g/day had an insignificant decrease in risk between 0.88 and 0.91 (hazard ratio (HR)). Groups above 32 g/day had significantly decreased risk between 0.68 and 0.81 (HR), although insignificant in subgroups sized below 1300.

At baseline, 7916 individuals reported never eating raw carrots during the previous 12 months. In total, 31,545 had a calculated consumption of less than 32 g/day and 16,414 individuals had a calculated consumption higher than 32 g/day. The incidence of CRC was 3.9% for those eating no raw carrots, 3.5% for those eating less than 32 g/day and 2.9% for those eating more than 32 g/day. *X*^2^-tests showed significant differences in the incidence of CRC according to raw carrot intake, gender, age groups, NSAID intake, BMI, and alcohol intake. No significant differences were seen according to previous cerebral or coronary artery thrombosis, MET score, all other vegetable intake or intake of other root vegetables (Table 1).

### 3.1. Comparison of High, Low and No Intake of Carrots

**Figure 2:**
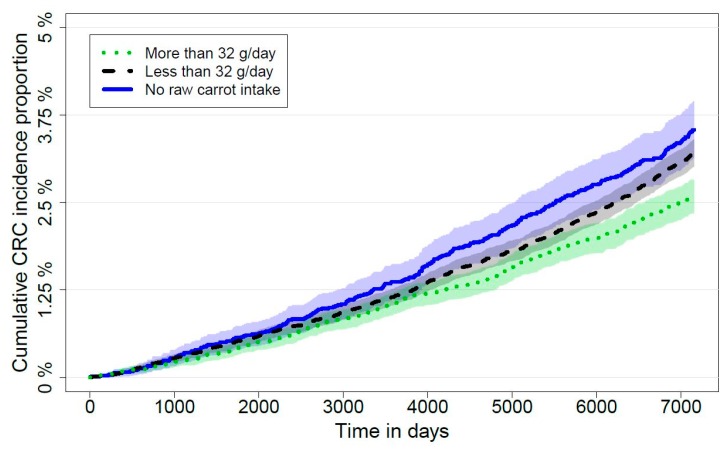
Cumulative incidence proportions of colorectal cancer (CRC) incidence according to self- reported raw carrot intake at baseline including 95% confidence intervals.

The cumulative incidence proportion of CRC was higher with a decreasing intake of raw carrots. The cumulative incidence proportions increased throughout follow-up for all three exposure groups (Figure 2).

**Figure 3:**
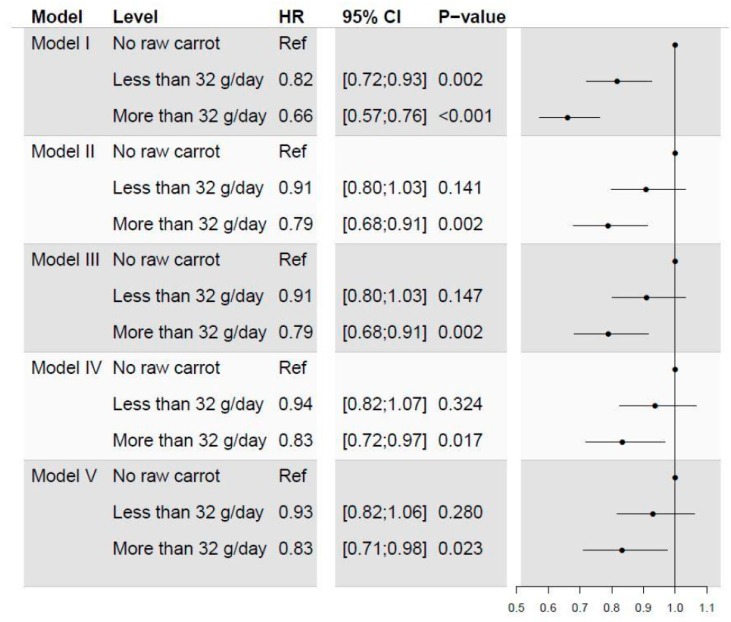
Forest plot visualizing the results of a univariate and four multivariate cox proportional hazard regression models estimating risk of CRC according to self-reported raw carrot intake. Model I: Univariate cox regression model. Model II: Adjusted for age group and gender. Model III: Further adjusted for previous cerebral or coronary artery thrombosis and nonsteroidal anti-inflammatory drugs (NSAIDs) intake. Model IV: Further adjusted for METs, BMI, smoking and alcohol intake. Model V: Further adjusted for other root vegetable intake and all other vegetable intake.

The univariate cox proportional hazard regression model (model I) showed significant decreases in risk of CRC in those with an intake less than 32 g/day (HR 0.82 CI95% 0.72; 0.93) and in those with an intake over 32 g/day (HR 0.66 CI95% 0.57; 0.76), compared to those with no intake of raw carrots. After adjusting for gender and age (model II) the risk differences were decreased and a significant difference were only seen in subgroup eating more than 32 g/day (HR 0.79 CI95% 0.68; 0.91). The subsequent adjustments (model III through V) had only minimal effect on the risk estimates for raw carrot intake. This resulted in a significant difference in risk for subgroup eating more than 32 g/day (HR 0.83 CI95% 0.71; 0.98), and an insignificant difference in risk for subgroup eating less than 32 g/day (HR 0.93 CI95% 0.82; 1.06), compared to subgroup eating no raw carrot, after full model adjustments (Figure 3).

### 3.2. Sensitivity Analyses

Sensitivity analyses showed that carrot juice, prepared/cooked carrots or total carrot intakes were not statistical significantly associated with incidence of CRC. Analyses of possible interactions between raw carrot intake and each of the covariates from the model V did not identify any significant interactions. Sensitivity analysis conducted using age as time resulted in similar HRs for raw carrot intake >32 g/day as did the main analyses. Risks of CRC were statistically significant at 0.71, 0.80, 0.80, 0.85 and 0.84 respectively in model I through V for those with an intake >32 g/day compared to those eating no raw carrot. Examination of Schoenfeld residuals verified the proportional hazards assumption.

## 4. Discussion

In this prospective cohort study, a population of 57,053 Danes was used to explore the association between dietary carrot intake and risk of CRC. The results showed that high carrot intake corresponding to >32 g raw carrot per day was associated with a 17% decreased risk of CRC, whereas an insignificant difference in risk of CRC was observed for those eating less than 32 g raw carrot per day, compared to those eating no raw carrot. To our knowledge, this is the first study evaluating the direct relationship between carrot intake and the incidence of CRC, although in a previous study using data from two case-control studies that included 1225 cases of CRC, it was shown that raw vegetables and in particular raw carrots caused risk reductions of 20% for CRC [60]. A preventive role of carrot in the development of CRC is plausible because carrots is the main dietary source of the bioactive polyacetylenic oxylipins FaOH and FaDOH, which have demonstrated anti-proliferative effects in cancer cells [25,29] and anti-neoplastic effects in CRC-primed rats in a dose-dependent relationship [21,23]. The preventive role of FaOH and FaDOH in relation to CRC is probably linked to their alkylating properties leading to covalent alkylation and inhibition of pro-inflammatory markers, enzymes, and inflammatory transcription factors that play an important role in cancer development and in particular CRC [21,61]. According to the preclinical rat studies, a cancer-preventive dose of polyacetylenes can be obtained, at an intake above 30 g of raw carrots per day, which furthermore substantiates the cancer-preventive effects of carrots as demonstrated in this cohort study.

Based on the numerous in vitro studies on the anti-proliferative effects of FaOH and FaDOH on cancer cells, it is clear that FaOH is more cytotoxic than FaDOH [21,25,29,30,31]. In addition, it has been shown that FaOH inhibits in vitro, the growth of the human epithelial colorectal adeocarcinoma cell line Caco-2, and that this effect is synergistically enhanced when combined with FaDOH [25]. In the recent studies on the anti-neoplastic effects of FaOH and FaDOH in CRC-primed rats the ratio of these polyacetylenes were 1:1 in the tested rat diets but other ratios may have resulted in even higher or lower CRC-preventive effects [21,23]. Thus, not only the concentration of FaOH and FaDOH in carrots may be important for the cancer-preventive effects of carrots but also the ratio between these polyacetylenes.

Factors that have an influence on the content of polyacetylenic oxylipins in consumed carrots, includes besides processing as mentioned in the introduction also storage and location of cultivation and not at least genotype. The cropping system on the other hand, i.e., organic versus non-organic cultivation does not affect the content of these bioactive constituents [45,54]. Consequently, there is a potential to optimize the content of polyacetylenic oxylipins in carrots and thus to increase the possible cancer-preventive effect of carrots and carrot products. There are considerable differences in the contents and ratio of FaOH and FaDOH between different carrot cultivars available in Denmark and on the marked in general. This complicate the picture in relation to the preventive effect of carrot intake on CRC but also show that there is a huge potential of optimizing the intake of FaOH and FaDOH in selecting carrot cultivars for human consumption with an optimal composition profile of these polyacetylenic oxylipins. Therefore, choosing the right carrot cultivar could increase the preventive effect on CRC. Bitterness is considered as an undesirable taste of carrots, which is primarily due to the content of FaDOH and di-caffeic acids that are mainly present in the peel and outer-layers of carrots [62]. Therefore, raw carrots are usually peeled before intake. Peeling not only changes the composition profile of FaOH and FaDOH of raw peeled carrots but also has an effect on the intake of these polyacetylenes because their concentration are higher in the peel per g fresh weight compared to the rest of the carrot, and this is particular the case for FaDOH. Thus, an optimal CRC-preventive effect of carrots not only depends on the cultivar but also how the carrots are processed before consumption.

Other apiaceous vegetables, such as celery, celeriac, fennel, parsley, and parsnip, do also contain FaOH and FaDOH and for some of these vegetables the concentrations of FaOH and FaDOH in the edible parts of these vegetables are even higher than in carrots [28,29]. However, these vegetables seems not to contribute significantly to the overall intake of polyacetylenes mainly because they are consumed in much lower quantities compared to carrots and because the root vegetables such as celeriac and parsnip are often subjected to thermal processing resulting in considerably losses of polyacetylenes as is the case for carrots. Intake of all other vegetables, including apiaceous vegetables, were found not to have any significant impact on risk of CRC because no other root vegetables nor all other vegetables were associated with incidence of CRC as demonstrated in model V (Figure 3). Hence, previous studies reporting lower cancer incidence with higher vegetable intake, may partially be due to the intake of carrots.

The study is based on self-reported 1 year recall of different food intakes and this is usually correlated with some bias. First of all, the self-reported recall will result in some bias as participants will have difficulties in remembering food intake as well as over-estimation of healthy food components [63,64]. Furthermore, carrot eaters may have a healthier behavior in general, although the adjustments for MET, other vegetable intake, smoking and alcohol intake did not affect the hazard ratios notably. If participants’ self-reported raw carrot intake is either randomly over- and underestimating actual intake, or systematically overestimating, the decrease in risk of CRC correlated with raw carrot intake is probably underestimated. Further, the true decrease in risk of developing CRC would probably be even greater if the type and handling of carrots were controlled.

In the food frequency questionnaire, the participants reported intake of raw carrots per week and in our study, effect on incidence of CRC was seen in intake as low as 2–4 carrots corresponding to >32 g raw carrot each day, i.e., half of a small-sized raw carrot each day. In the present study, the carrot cultivars were not reported. However, if we look at the contents of polyacetylenes in an average carrot, this will not give 24 h protection of the epithelial cells and so the effect of the carrots seems to be long term. The halftime of FaOH and FaDOH has been shown to be approximately 5–6 h in the blood circulation after intake of carrot juice [30,65,66].

Prepared/cooked carrots, carrot juice and total carrot intake were not statistical significantly associated with incidence of CRC as expected even though this may increase the bioavailability of bioactive polyacetylenic oxylipins. This is probably due to a great loss of FaOH and FaDOH in preparation or preserving methods as described in the introduction.

Participants differed from non-participants, as the non-participants as a whole had a lower socio-economic status than participants [48]. This would mean that the results may not be transferrable to the general population, although the possibility of polyacetylenic oxylipins affecting socio-economic strata in different ways is considered unlikely. This is also supported by the fact that no interactions were found between raw carrot intake and any covariate, even though health-affecting behaviors are often associated with socio-economic status.

## 5. Conclusions

Self-reported intake of raw carrot at a baseline of 2–4 carrots or more each week (>32 g/day) was associated with a 17% decrease in risk of CRC, with a mean follow-up of more than 18 years, compared to individuals with no intake of raw carrots even after extensive model adjustments. An intake below 2–4 carrots each week (<32 g/day) was not significantly associated with risk of CRC.

## Figures and Tables

**Table 1:** Baseline characteristics of individuals with and in individuals without colorectal cancer (CRC) incidence during follow-up, *n* = 55,875.

Variable	Level	CRC ^a^ *n* = 1889	No CRC *n*= 53,986	Total *n* = 55,875	*p*-Value
Raw carrot intake	None	306 (3.9) ^a^	7610 (96.1) ^b^	7916	
	0–32 g/day	1105 (3.5)	30,440 (96.5)	31,545	
	Over 32 g/day	478 (2.9)	15,936 (97.1)	16,414	<0.001
Gender	Female	857 (2.9)	28,373 (97.1)	29,230	
	Male	1032 (3.9)	25,613 (96.1)	26,645	<0.001
Smoking	Non-smoker	586 (3.0)	18,993 (97.0)	19,579	
	Former smoker	603 (3.7)	15,513 (96.3)	16,116	
	Current smoker	700 (3.5)	19,480 (96.5)	20,180	<0.001
NSAID intake	No	1347 (3.6)	36,311 (96.4)	37,658	
	Yes	542 (3.0)	17,675 (97.0)	18,217	<0.001
Body Mass Index	Normal	717 (3.0)	23,386 (97.0)	24,103	
	Low	16 (3.4)	459 (96.6)	475	
	High	1156 (3.7)	30,141 (96.3)	31,297	<0.001
Previous cerebral or	No	1830 (3.4)	52,278 (96.6)	54,108	
coronary artery thrombosis	Yes	59 (3.3)	1708 (96.7)	1767	0.974
Alcohol intake	Within recommendation	1006 (3.2)	30,918 (96.8)	31,924	
	No alcohol	41 (3.2)	1254 (96.8)	1295	
	Over recommendation	842 (3.7)	21,814 (96.3)	22,656	0.001
Age group	50–54 years	634 (2.7)	22,990 (97.3)	23,624	
	55–59 years	591 (3.4)	16,701 (96.6)	17,292	
	60–65 years	664 (4.4)	14,295 (95.6)	14,959	<0.001
Other root	1st quartile	478 (3.4)	13,457 (96.6)	13,935	
vegetables	2nd quartile	486 (3.5)	13,481 (96.5)	13,967	
	3rd quartile	496 (3.5)	13,489 (96.5)	13,985	
	4th quartile	429 (3.1)	13,559 (96.9)	13,988	0.116
All other vegetables	1st quartile	485 (3.5)	13,444 (96.5)	13,929	
	2nd quartile	481 (3.4)	13,500 (96.6)	13,981	
	3rd quartile	466 (3.3)	13,491 (96.7)	13,957	
	4th quartile	457 (3.3)	13,551 (96.7)	14,008	0.738
METs—h/week	1st quartile	498 (3.5)	13,721 (96.5)	14,219	
	2nd quartile	467 (3.3)	13,783 (96.7)	14,250	
	3rd quartile	466 (3.4)	13,073 (96.6)	13,539	
	4th quartile	458 (3.3)	13,409 (96.7)	13,867	0.677

^a^ CRC = Colorectal cancer ^b^ Values in parentheses are row percentages.

## Abbreviations

BMI	Body mass index
COX	Cyclooxygenase
CRC	Colorectal cancer
FaOH	Falcarinol
FaDOH	Falcarindiol
HR	Hazard ratio
MET	Metabolic equivalents
NSAID	Nonsteroidal anti-inflammatory drug
